# Autoimmune hepatitis complicated by adult‐onset Still's disease during treatment with tocilizumab: A case report from acute onset to recurrence

**DOI:** 10.1002/ccr3.7530

**Published:** 2023-06-29

**Authors:** Daiki Uchihara, Tomohiro Suzuki, Yudai Koya, Mayumi Tai, Osamu Ichii, Nobuo Matsuhashi, Yutaka Ejiri, Tomoya Kato, Yuichi Honma, Michihiko Shibata, Masaru Harada

**Affiliations:** ^1^ Department of Gastroenterology Fukushima Rosai Hospital Iwaki Japan; ^2^ Third Department of Internal Medicine, School of Medicine University of Occupational and Environmental Health Kitakyushu Japan; ^3^ Department of Pathology Fukushima Rosai Hospital Iwaki Japan

**Keywords:** adult‐onset Still's disease, autoimmune hepatitis, liver biopsy

## Abstract

The characteristics of liver dysfunction due to adult‐onset Still's disease are not specific. Differentiating from autoimmune hepatitis is important in deciding whether to continue corticosteroid therapy, and also in terms of management of cirrhosis and surveillance of hepatocellular carcinoma. Liver biopsy is thought to be the most important determinant for differential diagnosis.

## Graphical abstract

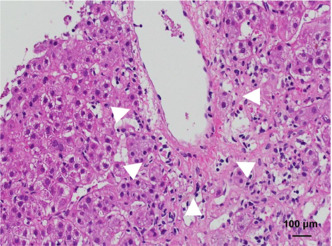

## 1 INTRODUCTION

Adult‐onset Still's disease (AOSD) is a systemic inflammatory disorder of unknown etiology that usually affects young adults.^1^ Spiking fever, arthritis, and evanescent rash are commonly observed during the course of the disease. Other frequently observed clinical features include sore throat, hepatosplenomegaly, lymphadenopathy, and serositis.^2^ Adult‐onset Still's disease is usually complicated by liver dysfunction.^3^ However, cases of autoimmune hepatitis (AIH) complicated by AOSD are rare, and discerning the cause of liver dysfunction as AOSD or AIH is difficult. This was a very rare case that could be followed up from acute onset to recurrence of AIH complicated by AOSD. Herein, we report a case of AIH complicated by AOSD that was successfully diagnosed by a liver biopsy.

## 2 CASE REPORT

A 31‐year‐old woman was diagnosed with AOSD in October 2013. The diagnosis was based on the presence of pyrexia, skin rash, sore throat, lymphadenopathy, leukocytosis, hyperferritinemia, and the lack of rheumatoid factor and antinuclear antibodies. The clinical findings met four major criteria (fever, arthralgia, typical rash, and leukocytosis) and three minor criteria (sore throat, lymphadenopathy, negative test for antinuclear antibody, and rheumatoid factor) for AOSD classification proposed by Yamaguchi et al.^1^ After the administration of 40 mg of prednisolone (PSL) per day, her symptoms improved and PSL was tapered. Prednisolone was maintained at 5–15 mg/day, depending on the severity of AOSD, but the patient's symptoms sometimes relapsed because of drug discontinuation. In October 2015, 50 mg/day cyclosporine A (CyA) was started, and the dose was increased to 100 mg/day in February 2017. In July 2017, tocilizumab was started as persistent fever, lymphadenopathy, and hyperferritinemia (3944 ng/mL) persisted. Doses of 360 mg (8 mg/kg) was administered every 2 weeks. The administration continued through October 2021.

**FIGURE 1:**
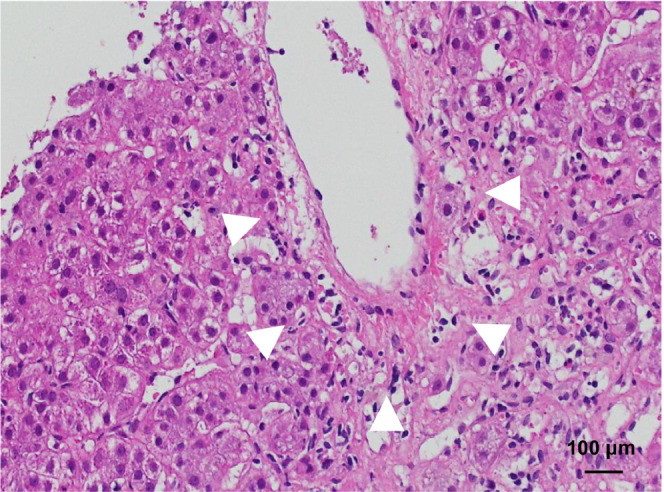
Hematoxylin–eosin staining of a section of the liver shows hepatocyte shedding around the central vein (arrowheads) and eosinophil infiltration at the time of the first acute liver injury in October 2017.

In October 2017, the patient was admitted to our hospital with liver dysfunction, which was the only finding, without any symptoms. Worsening of AOSD was not suspected as the C‐reactive protein (CRP) concentration was almost normal and there was only mild hyperferritinemia (Table 1). Liver biopsy revealed the collapse of hepatocytes around the central vein (Figure 1), infiltration of eosinophils, Kupffer cell hyperplasia, and mild fibrosis around the Glisson's capsule. We suspected liver dysfunction due to AOSD, acute‐onset AIH, or drug‐induced liver injury (DILI) caused by tocilizumab. We started methylprednisolone (mPSL) 125 mg/day for the first 3 days, followed by oral administration of PSL (50 mg/day) and CyA (100 mg/day). Prednisolone was gradually tapered and continued at 5 mg/day. However, in September 2020, both PSL and CyA were discontinued because of the patient's strong desire to stop taking them.

**TABLE 1:** Laboratory findings at the time of acute liver injury.

	October 2017/January 2021		October 2017/January 2021	
**Hematology**		**Serology**		
WBC	8000/5500 per μL	CRP	0.54/0.11 mg/dL	
Neutro	69/66.1%	Ferritin	1650/2202 ng/mL	
Lympho	19.3/20.6%	IgG	2536/2362 mg/dL	
RBC	488 × 10⁴/424 × 10⁴ per μL	IgA	344/280 mg/dL	
Hb	13.8/12.6 g/dL	IgM	145/74 mg/dL	
Plt	19.4 × 10⁴/17.5 × 10⁴ per μL	ANA	80/80	
		AMA	(−)/(−)	
**Biochemistry**		Hyaluronic acid	220/126 ng/mL	
AST	2267/1884 U/L	Type 4 collagen 7S	10/7.8 ng/mL	
ALT	1493/2072 U/L	HBs‐Ag	(−)/(−)	
LDH	899/671 U/L	HBV‐DNA	(−)/(−)	
ALP	513/371 U/L	HCV‐Ab	(−)/(−)	
T‐Bil	8.46/6.86 mg/dL	HIV‐Ab	(−)/(−)	
D‐Bil	6.38/5.41 mg/dL	CMV antigen	(−)/(−)	
γGTP	101/87 U/L	Rheumatoid factor	(−)/(−)	
BUN	7.4/6.5 mg/dL	P/C‐ANCA	(−)/(−)	
Cre	0.49/0.48 mg/dL	Ceruloplasmin	No record/31.6 mg/dL	
Alb	3.9/4.3 g/dL			
NH₃	21/91 μmol/L			
		**Cytokine markers**		**(Normal values)**
**Coagulation**		TNF‐α	No record/18.9 pg/mL	(2.27–11.2)
PT	104/62%	IL‐1	No record/0.2 pg/mL	(≦0.928)
		IL‐6	No record/3.43 pg/mL	(≦2.41)
		IL‐18	No record/67,500 pg/mL	(≦211)

Abbreviations: Alb, albumin; ALP, alkaline phosphatase; ALT, alanine aminotransferase; AMA, anti‐mitochondrial antibody; ANA, antinuclear antibody; ANCA, anti‐neutrophil cytoplasmic antibody; AST, aspartate aminotransferase; BUN, blood urea nitrogen; CMV, Cytomegalovirus; Cr, creatinine; CRP, C‐reactive protein; D‐Bil, direct‐bilirubin; Hb, hemoglobin; HBs‐Ag, hepatitis B surface antigen; HBV‐DNA, hepatitis B deoxyribonucleic acid; HCV Ab, hepatitis C antibody; HIV‐Ab, human immunodeficiency virus; Ig, immunoglobulin; IL, interleukin; LDH, lactate dehydrogenase; Plt, platelet; PT, prothrombin time; RBC, red blood cell; T‐Bil, total bilirubin; TNF‐α, tumor necrosis factor‐α; WBC, white blood cell.

**FIGURE 2:**
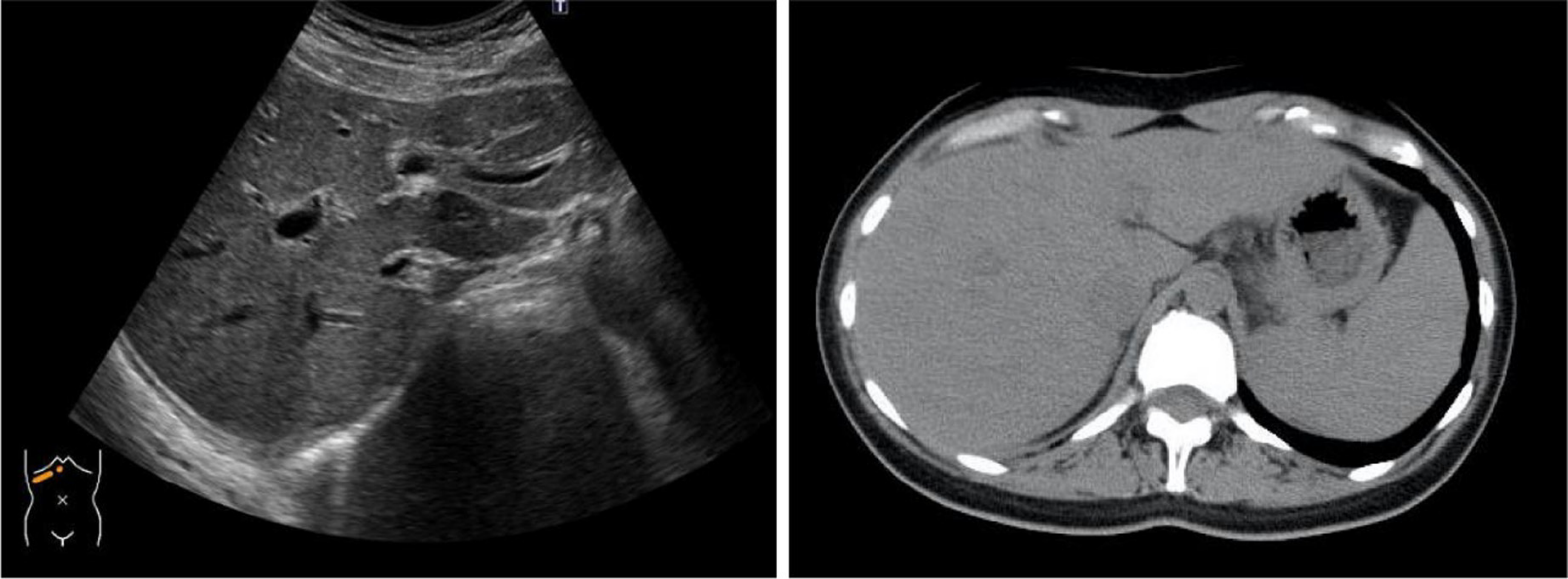
Abdominal ultrasound and computed tomography scans revealed mild hepatosplenomegaly and periportal edema. The echo levels of the liver are decreased and crude.

**FIGURE 3:**
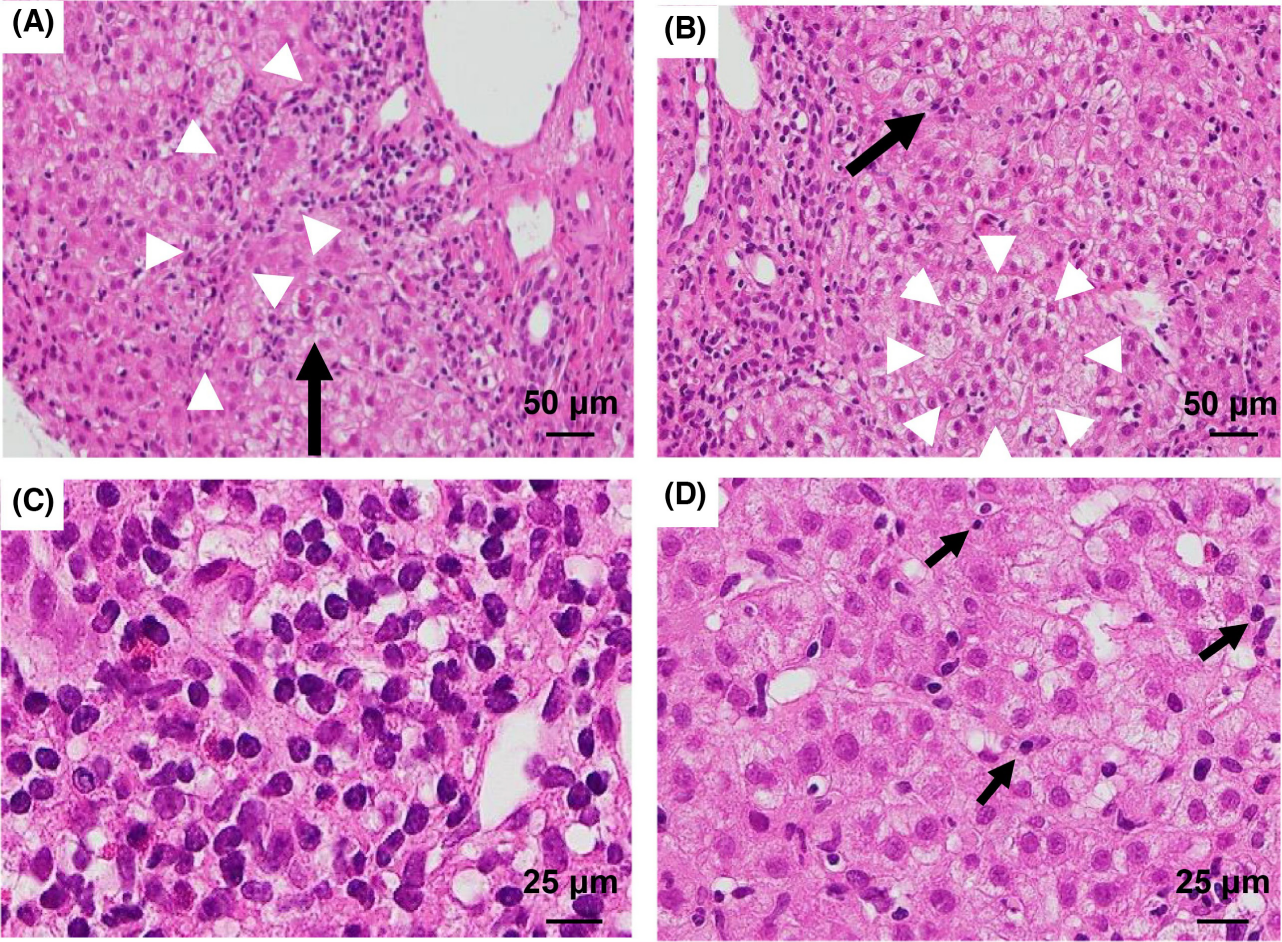
(A) Hematoxylin–eosin staining of a section of the liver shows interface hepatitis (surrounded by arrowheads) and piecemeal necrosis (arrow). (B) Rosette (surrounded by arrowheads) and hepatocyte ballooning (arrow) were also observed. (C) Abundant infiltration of plasma cells that have a round, eccentrically placed nucleus and a perinuclear halo is noted. (D) Emperipolesis (arrow), engulfment of lymphocytes (or other inflammatory cells) by hepatocytes, probably reflecting immune‐mediated injury, was also observed at the time of the second liver injury in January 2021.

**FIGURE 4:**
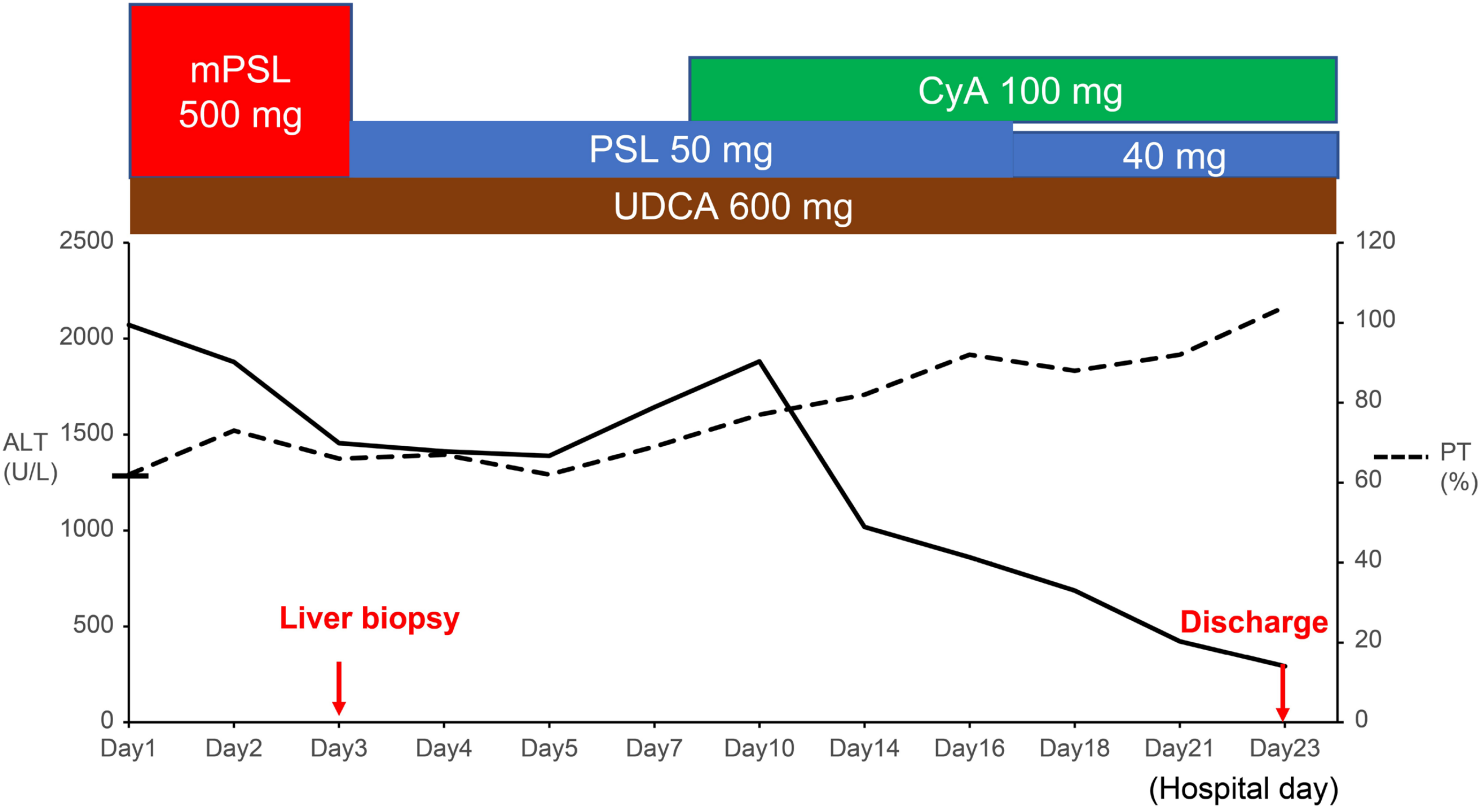
Evaluation of ALT and PT after the second onset of liver injury. Treatment with mPSL was initiated on the day of hospitalization, followed by maintenance with oral PSL 50 mg/day. Liver function was exacerbated on Day 8; therefore, 100 mg CyA was administered. ALT, alanine aminotransferase; CyA, cyclosporine A; mPSL, methylprednisolone; PSL, prednisolone; PT, prothrombin; UDCA, ursodeoxycholic acid.

Thereafter, she presented to our hospital in January 2021 with a history of fatigue, loss of appetite, and jaundice for 3 days. Physical examination revealed diffuse jaundice, but no lymphadenopathy or rash that suggested the worsening of AOSD. The laboratory findings are shown in Table 1. There were no specific results for other liver diseases, as of October 2017. Imaging revealed hepatosplenomegaly without tumorous or obstructive hepatobiliary diseases (Figure 2). Liver biopsy revealed interface hepatitis, hepatocyte rosette formation, periportal inflammation, plasma cell infiltrates, and emperipolesis; these are indicative of AIH (Figure 3). As her revised international AIH group score was 18, we diagnosed the patient with AIH complicated by AOSD. We started mPSL semi‐pulse therapy (500 mg/day) for the first 3 days, followed by a maintenance dose of oral PSL 50 mg/day in combination with ursodeoxycholic acid 600 mg/day. Her symptoms and liver dysfunction improved gradually, but liver function was exacerbated on Day 8. We therefore administered 100 mg of CyA, which improved liver function (Figure 4). Subsequently, PSL was tapered by 5–10 mg every 2 weeks. The patient's liver function is currently normal on a dosage of PSL 3 mg/day and CyA 100 mg/day.

## 3 DISCUSSION

Adult‐onset Still's disease is a systemic inflammatory disease that exhibits a bimodal age distribution, with the first peak age at 15–25 years and the second at 36–46 years.^3^ It is an auto‐inflammatory disorder and suggests the involvement of a pro‐inflammatory cascade. Several factors actively contribute to the amplified inflammatory response of AOSD, which is often expressed as a cytokine burst or storm.^4^ This inflammatory response causes various symptoms, such as fever, vanishing rash, and polyarthritis, and is sometimes complicated by liver dysfunction, which occurs in 85% of cases of AOSD.^3^ Autoimmune hepatitis has peak ages at 10–30 and 40–60 years, and 71–95% of adults and 60–80% of pediatric patients are predominantly female. Autoimmune hepatitis is a serious autoimmune liver disease characterized by the progressive destruction of the liver parenchyma and chronic liver fibrosis.^5^

Three cases of AIH complicated by AOSD have been reported previously (Table 2). The first and second cases^6^, ^7^ were simultaneous occurrences of AOSD and AIH, but an accurate diagnosis of AIH could not be made as liver biopsy was not performed. The third patient had AIH prior to AOSD.^8^ Therefore, our patient was the first to develop AOSD before AIH and is a very rare case that could be followed up from acute onset to recurrence of AIH complicated by AOSD.

**TABLE 2:** Previous reports of autoimmune hepatitis complicated by adult‐onset Still's disease and that of our patient.

Case	References	Age	Sex	Onset	Activity of AOSD	AIH score	Liver biopsy	Treatment	Progress
1	(8)	27	Female	Simultaneous	+	14–17	−	Corticosteroids Plasmapheresis	Relief
2	(9)	36	Female	Simultaneous	+	17	−	Corticosteroids Immunoglobulin	Death
3	(10)	18	Female	AIH → AOSD	−	16	+	Corticosteroids Methotrexate Infliximab	Relief
Our case		32	Female	AOSD → AIH	−	18	+	Corticosteroids Cyclosporine	Relief

Abbreviations: AIH, autoimmune hepatitis; AOSD, adult‐onset Still's disease.

It is sometimes difficult to distinguish AIH from AOSD. In a report on the clinical findings of 306 cases of rheumatic diseases with liver dysfunction, two‐thirds of the cases were diagnosed as definite or probable AIH, based on the diagnostic criteria of the International Autoimmune Hepatitis Group.^9^ However, a liver biopsy was performed in only 15 cases, and most patients showed no or mild fibrosis and minimal or mild activity. The use of an international scoring system without a liver biopsy may cause an over‐diagnosis of AIH. Histological features of liver biopsy samples from patients with AOSD include periportal mononuclear infiltration, Kupffer cell hyperplasia, lobular inflammation, and massive or sub‐massive hepatic necrosis.^10^ These findings are not specific and are often found in AIH or DILI. Ground glass‐like cytoplasmic inclusions, which are associated with venous outflow impairment, are also observed in AOSD.^11^ A typical feature of AIH is the presence of interface hepatitis, piecemeal necrosis, plasma cell‐rich infiltrates, and emperipolesis.^12^ Emperipolesis is the engulfment of lymphocytes by hepatocytes, probably reflecting immune‐mediated injury that occurs in several liver diseases (including AIH, chronic hepatitis B, and chronic hepatitis C).^13^

In the present case, acute liver injury occurred twice, once in October 2017, and again in January 2021. There were no typical symptoms of AOSD at either time point. During the first liver injury, histopathology revealed a collapse of hepatocytes around the central veins, an infiltration of eosinophils, and Kupffer cell hyperplasia. These findings evoked liver dysfunction of AOSD, acute‐onset AIH, and DILI due to tocilizumab, but the findings were not specific. The second liver injury had typical pathological findings of AIH. Viewed retrospectively, the first liver injury might have been acute‐onset AIH, and the second liver injury was AIH exacerbated by drug suspension for 4 months.

Regarding the first liver injury, it is controversial whether acute‐onset AIH occurred despite the patient undergoing immunosuppressive treatment for AOSD. Immunosuppressive treatment could make it difficult to detect the typical pathological findings of AIH. Pongpaibul et al. reported the characteristics of de novo AIH after liver transplantation in patients receiving immunosuppressive therapy.^14^ In 33% of the pretreatment biopsy samples in which AIH showed no acute cellular rejection, chronic rejection, or bile duct obstruction, there was no interface necro‐inflammatory activity. Furthermore, 10% of the biopsy samples obtained at the time of diagnosis had minimal nonspecific changes without interface or lobular necro‐inflammatory activity. Although tocilizumab‐induced severe liver injury has been reported as a DILI,^15^ tocilizumab‐induced AIH cannot be ruled out in the present case. Tocilizumab‐induced AIH has not yet been reported, but antitumor necrosis factor alpha (TNF‐α) agents can cause AIH.^16^ Tocilizumab, a humanized monoclonal antibody against the interleukin (IL)‐6 receptor, may cause a similar immune response leading to the onset of AIH.

We also focused on serological data, especially CRP and serum ferritin levels. The mean CRP levels are 11.3 ± 7.9 mg/dL in patients with AOSD.^17^ High serum ferritin levels may be observed in a variety of pathological conditions. Seventeen cases of severe hepatitis associated with AOSD have been reported, with 14 cases having serum ferritin above 3000 ng/mL and nine cases having serum ferritin above 10,000 ng/mL.^17^ In the present case, CRP was below 1.0 mg/dL and the serum ferritin levels were below 3000 ng/mL (Table 1), suggesting a low possibility of AOSD exacerbation.

Elevated serum cytokine levels are characteristic of both AOSD and AIH. Interleukin‐1, IL‐6, IL‐18, interferon‐gamma, and TNF‐α play a key role in the pathogenesis of AOSD, and elevated IL‐1 and IL‐18 levels are closely associated with the systemic symptoms of AOSD, such as fever, rash, and liver dysfunction.^18^ The IL‐18 and IL‐21 levels were significantly higher in patients with AIH than in those with other liver diseases and autoimmune disorders.^19^ In the present case, the serum IL‐18 levels (67,500 pg/mL) did not help in differentiating liver dysfunction as being due to AOSD or AIH. Further investigations are necessary to determine cytokine markers. One month after initial treatment with mPSL semi‐pulse therapy (500 mg/day), the serum IL‐18 levels decreased to 9050 pg/mL, which might reflect the therapeutic effect on AIH in the present case.

The first‐line treatment for AOSD and AIH is corticosteroids. Differentiating AIH from AOSD is important in deciding whether to terminate or continue corticosteroids. If the patient's condition is stable, corticosteroids can be terminated in about 40–50% of patients with AOSD.^2^ However, most patients with AIH take corticosteroids semi‐permanently as exacerbations are reported on discontinuing the treatment.^20^ At the time of the first liver injury, we could not confirm the diagnosis of AIH. Considering the possibility of AIH, we should have continued corticosteroids and followed up the patient closely. Treatment with CyA was effective for the second liver injury. Standard treatments for AIH are based on corticosteroids and azathioprine, and lead to disease remission in 80%–90% of patients.^5^ Cyclosporine A has been reported to be effective in AIH as a first‐line option or as a treatment for patients who do not respond to corticosteroids and azathioprine. Alternative first‐line treatment has been attempted with budesonide or CyA, but their superiority over standard treatment remains unclear.^5^ Due to the history of treatment with CyA without apparent side effects, we chose it as an additional treatment to corticosteroids.

In conclusion, in patients with AOSD, it is difficult to distinguish the cause of liver dysfunction as AOSD or AIH. We need to carefully evaluate the clinical symptoms, serological examination, and liver biopsy to determine appropriate treatment. Liver biopsy may be the most useful option for the differential diagnosis of AOSD and AIH. If AIH cannot be completely ruled out, corticosteroids should be continued to avoid the possible relapse of AIH.

## AUTHOR CONTRIBUTIONS

**Daiki Uchihara:** Conceptualization. **Tomohiro Suzuki:** Conceptualization. **Yudai Koya:** Conceptualization. **Mayumi Tai:** Conceptualization. **Osamu Ichii:** Conceptualization. **Nobuo Matsuhashi:** Conceptualization. **Yutaka Ejiri:** Conceptualization. **Tomoya Kato:** Conceptualization. **Yuichi Honma:** Conceptualization. **Michihiko Shibata:** Conceptualization. **Masaru Harada:** Conceptualization.

## FUNDING INFORMATION

None.

## CONFLICT OF INTEREST STATEMENT

The authors declare that there are no conflicts of interest.

## CONSENT

Written informed consent was obtained from the patient to publish this report in accordance with the journal's patient consent policy.

## ETHICS STATEMENT

This study did not include experiments on animals or humans.

## PATIENT CONSENT

The patient gave consent to publish her details in this case study.

## Data Availability

The data that support the findings of this study are available on request from the corresponding author. The data are not publicly available due to privacy or ethical restrictions.
